# Factors influencing fall prevention for patients with spinal cord injury from the perspectives of administrators in Canadian rehabilitation hospitals

**DOI:** 10.1186/s12913-019-4233-8

**Published:** 2019-06-17

**Authors:** H. Singh, B. C. Craven, H. M. Flett, C. Kerry, S. B. Jaglal, M. P. Silver, K. E. Musselman

**Affiliations:** 1 SCI Mobility Lab, Toronto Rehabilitation Institute – University Health Network, 520 Sutherland Dr, Toronto, ON M4G 3V9 Canada; 2 Rehabilitation Sciences Institute, University of Toronto, Toronto, Canada; 3 Division of Physical Medicine and Rehabilitation, Faculty of Medicine, University of Toronto, Toronto, Canada; 4 Institute of Health Policy, Management and Evaluation, University of Toronto, Toronto, Canada; 5 Department of Physical Therapy, Faculty of Medicine, University of Toronto, Toronto, Canada

**Keywords:** Health policy, Fall prevention, Interpretive description, Administrators, Spinal cord injury, Rehabilitation hospital

## Abstract

**Background:**

Fall prevention is a priority in Canadian tertiary rehabilitation hospitals. We aimed to understand the perspectives of hospital administrators on the challenges experienced when implementing fall prevention policies/procedures for patients with spinal cord injury (SCI) in tertiary rehabilitation hospitals.

**Methods:**

Semi-structured interviews were conducted with 10 administrators employed in six Canadian tertiary rehabilitation hospitals. Guided by an interpretive description framework, interviews were analyzed using a constant comparison approach.

**Results:**

Challenges with fall prevention experienced by administrators fell into the three categories: 1) fall prevention policy and procedural challenges (e.g. fall prevention policy not SCI-specific, expectation of zero falls, determining contributing factors, learning from falls, and overall effectiveness of the fall prevention policy), 2) clinician-related challenges (e.g. variable staff adherence with the organizations’ fall prevention procedures, inconsistent delivery of fall prevention education, and integrating individualized fall risks to guide clinical practice), and 3) patient-related challenges (e.g. balancing risk vs independence and rehabilitation progress, responsibility for fall prevention, and non-preventable falls).

**Conclusions:**

Fall prevention policies/procedures required by the hospitals were insufficient for clinical practice in SCI rehabilitation.

**Electronic supplementary material:**

The online version of this article (10.1186/s12913-019-4233-8) contains supplementary material, which is available to authorized users.

## Background

Fall prevention is a top priority in Canadian hospitals [1–3]. Accreditation Canada (2017) mandates hospitals implement evidence-based fall prevention procedures to reduce falls within healthcare settings. Fall prevention procedures in Canadian hospitals should include a fall risk assessment, documented and coordinated fall prevention approach, as well as methods for evaluating and modifying the fall prevention approach [1].

Hospital falls can result in liability, increased patient length of stay, injuries, delayed rehabilitation [4, 5], and greater care costs [6, 7]. In Canada, hospital falls are a safety concern [2] that are tracked and reported as hospital harm data [8].

Support from leadership staff can increase the success of fall prevention in hospitals [9]. Hospital administrators are responsible for implementing research-based clinical practices [10] and creating a safety culture [11]. A qualitative case study explored how leadership staff (*n* = 95) implemented the Registered Nurses’ Association of Ontario’s Falls Best Practice Guidelines in three Canadian acute care hospitals. Recognizing the perspectives of point-of-care nursing staff and having a simple implementation process were among two of the four fall prevention recommendations proposed [12]. However, this study lacked an inter-professional perspective as most participants had a nursing background [12]. Watson, Salmoni, & Zecevic (2018) identified the following determining factors to falls on neuroscience and medicine units: inadequate hospital policies, lack of staff education, and the patients’ cognitive/mobility issues [13]. However, findings from these aforementioned studies are limited to acute care settings; thus, they may not be relevant to rehabilitation hospitals. Nonetheless, these studies provide insight into important challenges with fall prevention implementation in Canadian acute care hospitals and highlight a need to explore fall prevention in tertiary rehabilitation hospitals.

Fall prevention policies and procedures in tertiary rehabilitation hospitals are often based on evidence from acute care settings [14] and studies with older adults [3, 15]. The application of acute care fall prevention strategies may not be appropriate in a rehabilitation context due to the differences in patient care goals [16]. In rehabilitation settings, the implementation of evidence-based fall prevention strategies is low [17] and conclusive evidence showing the effectiveness of hospital fall prevention programs is lacking [18]. Research on falls in rehabilitation units indicates that patients with neurologic disorders such as spinal cord injury (SCI) have a higher risk of falling in comparison to “orthopedic, cardiac, pulmonary disorders, prolonged stay on medical or surgical units, or trauma without spinal cord injury or head injury” [14]. The rate of falls among patients with SCI in inpatient rehabilitation is 13% [14]. Differences in fall rates between hospital units with similar patient populations have been reported as well [15]. Therefore, both the patient population and individualized risk assessments should be considered when implementing fall prevention strategies in tertiary rehabilitation hospitals [2, 19, 20]. In addition to the lack of an inter-professional perspective on fall prevention in rehabilitation hospitals [12], there are no SCI-specific fall prevention screening and guidelines for rehabilitation hospitals.

## Aim

Administrators offer a valuable perspective on fall prevention as they play a vital role in creating a culture that promotes patient safety and can influence buy-in from clinical staff [11, 21]. In this study we sought to understand the perspectives of hospital administrators who provide services to patients with SCI in tertiary rehabilitation hospitals throughout Canada on the challenges experienced with fall prevention.

## Method

### Design

This qualitative study was guided by the interpretive description framework [22]. Interpretive description was suitable for this study because it helped to generate clinically grounded results that can guide improvements in clinical settings [22]. Ethical approval for this study was obtained from the Research Ethics Board of the University Health Network (UHN).

### Settings and participants

A study using interpretive description can be conducted with various sample sizes, but is typically conducted with samples that range between 5 and 30 participants [22]. Taking into consideration the purpose of our study and the number of tertiary rehabilitation hospitals that serve Canadians with SCI (*n* = 15), we aimed to recruit 10 administrators from different rehabilitation units to generate, “a new and richly textured understanding” [22, 23]. Purposeful snowball sampling [24] was used as we sought to recruit individuals with specific occupational roles (e.g. administrators) and skills (e.g. fall prevention). Inclusion criteria required administrators to 1) work in a Canadian tertiary rehabilitation hospital with designated SCI beds, 2) provide services to patients with SCI, and 3) be responsible for creating, amending, and/or implementing the tertiary rehabilitation hospital’s fall prevention policies.

### Data collection

Semi-structured interviews were conducted November 2017 to April 2018 in person (*n* = 3) or by telephone (*n* = 7) by the lead author (HS), who is an occupational therapist with qualitative research and clinical experience in SCI rehabilitation. The interview questions queried perspectives on fall prevention, education, and post-fall management in SCI rehabilitation, and transfers of fall management responsibility from hospital staff to the patient/caregiver [25] during discharge from SCI rehabilitation (see Additional file 1 for the interview guide). Interviews were audio-recorded (Olympus-WS852) and lasted between 30 and 60 min.

### Analysis

In line with an interpretive descriptive approach, data collection and analysis occurred concurrently [22]. Audio-recorded interviews were transcribed verbatim by HS using Dragon Dictation [26] transcription software. The researchers used NVivo 12 (QSR International Pty Ltd.) to organize and analyze the data. Three research team members (HS, CK, KEM) independently reviewed the transcripts and discussed their interpretation of the data. Taking an interpretive description approach, we used inductive, open coding to analyze large sections of data. Analysis involved repeated immersion in the data. To understand the broader concepts and relationships in the data, we repeatedly asked ourselves “What is happening here?” and “What am I learning about this?” [27]. Codes were developed for the first transcript and subsequent transcripts were compared to the previous transcripts, using the constant comparative approach to analysis [28]. Conceptual diagrams [22] were created to understand how concepts related to each other. Upon analysis of all transcripts, a co-author (HMF) - who is an administrator in a SCI rehabilitation hospital with 12 years of experience and not a study participant – performed a “thoughtful clinician test” [22]. This allowed us to verify that our interpretations of the collected data “are plausible and confirmatory” [22].

## Results

Ten administrators (two males) from six different Canadian tertiary rehabilitation hospitals participated in this study (see Table 1). Most administrators had a varied interdisciplinary clinical background e.g. physiotherapy (3), social work (1), occupational therapy (1), exercise therapy (1), nursing (2), and medicine (1).

**Table 1:** Administrators, setting and rehabilitation unit type

Participant	Setting	Rehabilitation unit type
A1-Site A	Inpatient	SCI-specific
A2-Site A	Outpatient	SCI-specific
A3-Site A	Inpatient	SCI-specific
A4-Site A	Inpatient + outpatient	SCI-specific
A5-Site B	Inpatient	General rehab
A6-Site D	Inpatient	General rehab
A7-Site E	Inpatient	Neurological rehab
A8-Site C	Inpatient	General rehab
A9-Site C	Inpatient	General rehab
A10-Site F	Inpatient	SCI-specific

The three main categories of challenges experienced by administrators implementing fall prevention in SCI rehabilitation included: 1) fall prevention policy and procedures, 2) clinicians (i.e. members of the multidisciplinary clinical care team, such as nurses, physicians, physical and occupational therapists), and 3) patients (Table 2).

**Table 2:** Categories, subcategories and supporting quotes

Quotes for Category 1: Fall prevention policy and procedural challenges	
Subcategory 1a Fall prevention policy not SCI-specific	“The policy tends to be generic for a bigger audience right? … Acute care, it is so different than rehab and the purpose of acute care is different. So I think it’s finding how we fit within the policy and how to manage within it.” (A2) “I do find it’s difficult with our clientele because I find a lot of what is in the policy and procedures seems to be based on people with cognitive deficits rather than someone with a spinal cord injury.” (A9)
Subcategory 1b Expectation of zero falls	“I don’t think we will never have any falls. I do think trying to have zero serious outcomes from a fall is a pretty good thing to work on. But given that we are rehab, the chances are we will have patients falling because they are trying to ambulate and they are trying to go back to the community.” (A3) “A few years ago, there was a real push on zero falls … Despite our best efforts we can’t hundred percent eliminate falls but we certainly can reduce falls and prevent injuries.” (A6)
Subcategory 1c Determining contributing factors	“I think finding precise and good information is sometimes difficult because it does happen that we find the patient on the floor. So it’s more difficult to document what happens in those situations...we do include falls that occur during physiotherapy but we are questioning that because there could be falls that, there are risks within the fact that we do rehabilitation so actually we consider those falls also but we are considering to take them out.” (A7) “The greatest challenge that we have is that we are not actually present for most of the falls … we generally find the patient on the floor as opposed to being involved in a situation where the patient then falls.” (A5)
Subcategory 1d Learning from falls	“My hope is that people look at it as a learning opportunity and preventing the likelihood of that happening to someone else. They could be more defensive or there could also be like a punitive approach rather than a learning approach.” (A4) “I think for us it is important to try to find the root cause and then try to avoid it because we don’t want people falling and hurting themselves.” (A2)
Subcategory 1e Overall effectiveness of the fall prevention policy	“Does the screening actually result in less falls. I don’t know. Is that actually necessary?” (A2) “Based on all of that scientific literature that is out there around falls there hasn’t been very good evidence around any interventions that truly prevent falls.” (A4)
Quotes for Category 2: Clinician-related challenges	
Subcategory 2a Variable staff adherence with the organizations’ fall prevention procedures	“We can see sometimes a very glaring gap around ‘Oh the STRATIFY has not been done yet’ and then next week ‘Oh the STRATIFY has not been done yet’ and next week same thing...Why is my staff continually week after week not filling out the falls risk assessment but my patients are not falling?” (A1) “I would probably even say 100% [adherence], [clinicians] know what their duties are in fall prevention and management.” (A8)
Subcategory 2b Inconsistent delivery of fall prevention education	“Maybe there is a gap in something we’re forgetting to tell them or teach them before they go home. So if there is some sort of linkage as to what’s actually happening in the community when somebody leaves.” (A3) “We don’t always do the teach back around patient family education … Do our patients truly understand this undertaking when they are trying to keep mobile and do they have a harm prevention strategy?” (A4)
Subcategory 2c Integrating individualized fall risks to guide clinical practice	“Stop rubberstamping and really try to figure out how we can hone staff skills at being better at anticipating and understanding risk factors. So train them in identifying as opposed to just filling the paper that they will never look at in the chart.” (A1) “I would say the only thing with tracking falls is when people are completing the paperwork … is it just the filling out a form or are people actually putting sort of the thought process into the root cause? So that analysis piece. But in terms of tracking of falls themselves, I think that we do fairly well with actually tracking numbers and injuries... I would say that would be one of the challenges - getting it to be more proactive about preventing falls in the first place.” (A6)
Quotes for Category 3: Patient-related challenges	
Subcategory 3a Balancing risk vs independence and rehabilitation progress	“They are working with the experts who were trained to help the person manage that. So they are always in a situation where they’re in a harness or there’s protective equipment to protect from an injury. Again, they are pushing themselves. That’s the whole point of rehabilitation. You’ve gotta push yourself outside of the limits.” (A5) “We are not going to stop them from transferring just because we want to keep them from falling. Because like a 20-year-old, this is going to be their life so...they have to learn if they do fall what they’re going to do about it...I almost think our patients are more of an exception to the rule.” (A9)
Subcategory 3b Responsibility for fall prevention	“They should be part of it [fall prevention] so then it doesn’t necessarily necessitate any sort of transfer [of responsibility]. They need to be in it from day one if they are doing rehab. They should be told this is what I need to watch for. This is what I need to do … it’s my accountability to check the brake … It’s not the nurse doing everything for them.” (A1) “We have to make sure our patients become experts of their own condition so they can recognize potentially risky situations for them.” (A10)
Subcategory 3c Non-preventable falls	“I think that the cause of the falls are actually related to people that don’t respect what they have been told so that is a challenge. We are not in total control of all the factors. When the client decides to not listen with what has been told, it is more difficult to control their risks or prevent falls I would say.” (A7) “If someone is learning to do transfers and they have a spasm and they are fatigued they might fall to the floor...he’s learning to do it … he did it with two nursing staff but then his knee buckled and he went to the floor, and we had to catch him. But we are practicing techniques that are taught to him. He was well supervised. He was using the right equipment, but it was just one of those unpredictable events.” (A9)

### Category 1: fall prevention policy and procedural challenges

The challenges related to the implementation of a fall prevention policy and procedures described by administrators included five subcategories: a) fall prevention policy not SCI-specific, b) expectation of zero falls, c) determining contributing factors, d) learning from falls, and e) overall effectiveness of the fall prevention policy.

#### 1a) Fall prevention policy not SCI-specific

All administrators questioned the applicability of their unit’s “generic” fall prevention policy and procedures as some parts were irrelevant to patients with SCI.

I find a lot of what is in the policy and procedures seems to be based on people with cognitive deficits rather than someone with a spinal cord injury, who has an incomplete injury, who is actually starting to mobilize and starting to transfer (A9).

One administrator explained how their unit made efforts to “[find] how we fit within the policy and how to manage within it” (A2). To enhance the utility of the fall prevention tools, it was explained that the tools should be clinician-led, “practical … visual, in your face, tells you about the patient situation in that moment, rather than onerous paperwork” (A1). Administrators recommended that the chosen tools should address the specific fall risk factors experienced after a SCI. It was also explained – in spite of the fall prevention policy recommendations – that the risk assessment tools used were not appropriate for patients with SCI. Similarly, an outpatient administrator on a SCI-specific unit explained, “we found through our data that almost all our patients were classified as a fall risk” (A2). This administrator further explained that the fall risk assessment process was modified for outpatients as it lacked useful information for clinicians. The outpatient team had also adopted a blanket approach to fall prevention.

#### 1b) Expectation of zero falls

It was explained that the way in which the organizations tracked falls suggested zero falls were expected by the organization. For instance, all falls were regularly tracked in each unit, regardless of whether an injury occurred. In some units, falls were tracked daily on boards posted in the unit. These boards were used as communication tools to inform staff of the numbers of falls that occurred on the unit. Further, each hospital received a “score card” where a hospital’s score reflected the number of patient falls that occurred in the hospital. However, administrators explained that the activity-based nature of rehabilitation made a goal of zero falls unrealistic to work towards. All administrators agreed that, “zero [incidents of falls] is what everyone wants but it’s not realistic” when working with patients with SCI in a rehabilitation hospital. Rather, the number of injurious falls “should be as low as possible” (A9). In the current study, administrators expected a five to 25% fall rate in SCI rehabilitation. Rather than striving for zero falls, administrators instead proposed two realistic goals that sought to: i) “reduce [the possibility of] falls and prevent injuries” (A6) and ii) “have zero serious outcomes from a fall” (A3).

#### 1c) Determining contributing factors

Administrators explained that it was their responsibility to complete follow-up investigations of each fall to determine whether a fall could have been prevented and classify the fall. Since many patient falls were not witnessed, categorizing and determining the root cause of a fall was difficult. Administrators suggested that clinicians could consult patients on their perspectives about the cause of the fall to gain a clearer understanding of how to address this issue. For some, challenges with categorization were also attributed to a lack of clarity as to what could be defined as a preventable or non-preventable fall.

Each unit’s fall prevention policy required reporting of all falls including, “no harm therapy falls”. These were falls that occurred during therapy, but did not result in injury. Although there were reported advantages of tracking no injury therapy falls, such as documenting for liability and: “to see what we could do differently … perhaps the patient wasn’t ready to participate in therapy that day” (A3). Not all administrators agreed with their organization’s requirement to report every fall. One administrator contemplated excluding mandatory reporting of therapy falls that did not result in an injury on his/her unit due to the process being overly time consuming for clinical staff.

#### 1d) Learning from falls

Administrators described barriers to learning from patient falls on a unit as including concerns that it left some staff feeling “defensive” about falls. Other concerns included the perception that some administrators had a “punitive” rather than a “learning” approach to reporting falls within the unit (A4). Additionally, the lack of “resources to complete well run debriefs” was another barrier to learning from falls (A4). Moreover, falls that occurred while a patient was off-site on a community pass were generally not tracked, but were viewed as opportunities to educate the patient. “Tracking falls that occur on the weekend gives the clinicians a chance to see whether the patient needs more education on specific risk factors or needs to be re-trained on how to do something like a transfer” (A1).

Administrators asserted that they reviewed incident reports pertaining to their units, but some questioned whether a larger review of current trends occurred. As depicted by A5, analyzing the trends of falls was viewed to be important for guiding improvements to fall prevention:

The other critical aspect is…the review of falls not only with staff on the unit at the time, but a larger review in general to look at what are the trends that we see and building prevention programs from that data (A5).

#### 1e) Overall effectiveness of the fall prevention policy

Two views were prevalent about the effectiveness of fall prevention in SCI rehabilitation. One view was since fall rates were “low” in SCI rehabilitation, fall prevention was seen to be effective. These administrators believed the aim and outcomes of fall prevention in SCI rehabilitation were to prevent falls only while the patient was in rehabilitation. The second view was that since fall rates were considerably higher in the community, some administrators questioned whether rehabilitation was adequately preparing people for falls in the community.

We can help train and educate them before they’re discharged to make it a better situation in the community … typically when somebody leaves unless we hear something from outpatients, we wouldn’t really know what’s happening to that patient until maybe they are readmitted to acute care (A3).

A larger research gap was recognized by some administrators with respect to the evidence available for both fall prevention for individuals with SCI, and effective fall prevention interventions in general. While universal precautions were considered useful strategies to prevent falls, there was discussion that more useful strategies were needed to effectively prevent and prepare patients with SCI for fall risks in the community. An administrator involved in quality improvement highlighted that limited research existed on effective strategies to reduce falls after someone sustains a SCI. This administrator suggested a future direction for SCI fall prevention research: “the field (is) to focus on other things like improving mobility, reducing injury as a result of falling and … co-creating care plans with patients and their families” (A4).

### Category 2: clinician-related challenges

Clinicians were viewed as the experts when it came to fall prevention. However, three specific clinician-related challenges with respect to fall prevention procedures were identified. Administrators experienced challenges with: a) variable staff adherence with the organizations’ fall prevention procedures, b) inconsistent delivery of fall prevention education, and c) integrating individualized fall risks to guide clinical practice.

#### 2a) Variable staff adherence with the organizations’ fall prevention procedures

Inconsistent staff adherence with fall prevention procedures within some rehabilitation/SCI units were reported. It is important to note this challenge was not experienced by all administrators, as some administrators reported excellent adherence with all aspects of their fall prevention procedures but questioned relevance. According to the administrators, a unit’s fall prevention policy mandated that all falls must be reported; however, this did not always occur in practice. Although administrators recognized that clinicians “try their best”, one identified area of non-adherence was related to tracking of the no injury therapy falls.

I wouldn’t be surprised if we have more falls than are documented in therapy...the organization wants it for every fall. I think that’s where we struggle because I think the therapists feel often like it … was more a controlled fall than a fall (A2).

The challenges associated with reporting therapy falls were believed to be a result of the clinician’s lack of time, and a blurring of when falls were a part of therapy versus when they were not. A4 explained that another reason for clinician’s underreporting related to “attitudes that falls are completely not preventable and that it’s a natural course of a spinal cord injury”. Since falls were common during therapy, it was suggested that clarification of which falls were to be reported during the therapy would be beneficial to improve adherence with reporting. An administrator opined that ultimately it was the manager who was responsible for creating a positive staff safety culture and influencing staff adherence with fall prevention procedures.

Another identified area of non-adherence concerned completion of the unit’s fall risk assessment. As A10 described, “what was difficult for my team [was] to do it on day one because they might not find that was the right moment to do it”. A1 hypothesized the reason for the clinician’s non-adherence could be due to a “resource disconnect” because, “we have asked them to do a process that is very onerous” (A1). In contrast, A3 – an administrator who worked in the same facility but on a different SCI-specific inpatient unit – reported that clinicians were complying with fall prevention procedures. In their views, A9 and A10 explained that non-adherence was justified in some cases because the available fall prevention tools or procedures were not applicable to the SCI population or the unit. As A9 explained, “I can’t always justify following the procedure to the letter because I don’t think it could be applied to all of our patients”.

#### 2b) Inconsistent delivery of fall prevention education

Inconsistent delivery of fall prevention education was noted by most administrators. All but one administrator explained that after a patient with SCI experienced a fall, there was a post-fall staff huddle that takes place. The post-fall huddle was a discussion of the details of a patient’s fall and identification of additional fall prevention strategies. It was reported that clinicians inconsistently conducted thorough follow-ups with patients after the huddle. Administrators explained that patients received some fall prevention education, but were unclear about the level of detail and whether it occurred consistently with all patients. The lack of consistent delivery of patient education was believed to result in patients being less prepared for their risk of falls in the community after being discharged.

There was no formal process for fall prevention education. A structured discussion centering fall etiology/cause and how to prevent future falls was viewed as a beneficial part of a patient’s rehabilitation. In order to improve patient education, one unit planned to add a patient educator to their team.

Fall prevention education was generally not a top priority in outpatient rehabilitation because, “they are maybe with us for 30 minutes” (A2). The outpatient manager explained that the patient’s fall history was only brought up by staff, “at the beginning of the first four sessions” or when, “patients sometimes bring it up when they come in” (A2). The outpatient administrator assumed that those who attend outpatient SCI rehabilitation had already been provided with the appropriate fall prevention education while an inpatient. In contrast, some inpatient administrators strongly believed that fall prevention education for community fall risk factors should be the responsibility of outpatient rehabilitation. “Structured” fall prevention education should be provided at discharge from inpatient units with a focus on ensuring, “the patients are well aware and competent to make the decisions on risks of mobility and the outcomes of potential falls” (A4). According to A4, efforts should also be made to ensure clinicians had “the best practice skills around teaching”, were aware of “their roles as teachers”, and equipped with skills to “deliver education to different kinds of learners”.

#### 2c) Integrating individualized fall risks to guide clinical practice

Due to the clinicians often using a “blanket approach to intervention,” which meant applying universal fall prevention strategies rather than tailoring fall prevention strategies to a patient’s specific risk factors, administrators found it was challenging to anticipate falls. During a patient’s inpatient stay, it was common practice for the fall risk assessment to be completed at admission but it was often only reassessed if the patient had fallen. Administrators believed that a patient’s fall risk assessment score was not meaningfully used by clinicians to inform individualized fall prevention plans. As A6 explained: “the challenges are for staff not to complete just a form … I don’t know if people are thinking through those individual risk factors and coming up with strategies for those” (A6). In terms of the format of the risk assessment tools, administrators indicated that although a checklist format streamlined documentation for clinicians, it was a barrier to critically thinking about patients’ unique risk factors. It was suggested that the fall risk assessment form be reformatted to allow, “for more problem-solving and [to make it easier to] look at individualized risk factors, and then care planning around those” (A6). Administrators felt that creating customized and individualized fall prevention plans was an area for improvement. An inter-professional team approach to fall prevention was also seen as important to, “really understand what the root causes of a patient’s risks for falls are and then mitigating those [risk factors]” (A4). Two particular units adopted an inter-professional team approach to fall prevention: “a physiotherapist and occupational therapist and also the nurse, they all do the assessment together, and then after that, they put in recommendations” (A7).

Another challenge identified with respect to individualizing fall prevention policies/procedures included determining the most appropriate time to provide patient education, and how to tailor it to the patients’ post-discharge environments. A recommendation for individualizing fall prevention plans was to create a formal dialogue with patients on their specific fall risk factors in rehabilitation and the community.

### Category 3: patient-related challenges

Administrators described patient-related challenges that made it difficult to successfully implement fall prevention procedures. Patient-related challenges included: a) balancing risk vs independence and rehabilitation progress, b) responsibility for fall prevention, and c) non-preventable falls.

#### 3a) Balancing risk vs independence and rehabilitation progress

Administrators agreed that rehabilitation presented an inherent risk of falls for patients as they attempted to regain their mobility and autonomy, and prepare for community reintegration. During rehabilitation sessions patients were: “pushing their limits and they want to see what they can do. They [also] want to see what they can’t [do] yet” (A5).

A tension between adhering to fall prevention policies/procedures, while simultaneously allowing patients to re-learn their physical abilities and improve their mobility was apparent. Administrators recognized the challenges patients faced as they were, “very independent prior to their injury … They want to try to get that sense of control back*”* (A5)*.* Falls that occurred during therapy were viewed as safer, better controlled, and supported. Therapy falls gave patients the opportunity to learn fall prevention and self-management skills considered instrumental for community reintegration.

Balancing the autonomy of a patient was important during the rehabilitation process. Some administrators felt the current policies and procedures were not clear on how staff should respond to situations where a patient did not want to comply. Below, A3 recounted a complicated and delicate situation. Here, staff were doing their best to follow the fall prevention procedures after a patient fell, but the patient was eager to get up from the fall:

“The patient was insisting I’m fine, I need to get up. We are saying no, no, we need to do this assessment first … At what point do we say we listened to what the patient has to say before we do all of our safety checks?” (A3).

#### 3b) Responsibility for fall prevention

In the hospital settings, clinicians were responsible for any activities related to fall prevention. Within some inpatient units, there was an apparent transfer of responsibility whereby the responsibility for fall prevention activities were transferred to the patient upon discharge. Administrators stressed that preparing patients to recognize and prevent fall risks in the community through providing fall prevention education was paramount. However, given a clinician’s lack of time and other priorities, this could be overlooked. One administrator explained to effectively prepare patients on how to manage and mitigate fall risk factors in the community, “we need to look at the model of care differently” (A1). Instead of clinicians conducting all fall prevention activities, clinicians should share this responsibility with patients. Shared responsibility for fall prevention was surprisingly only described by administrators from two inpatient units. Clinicians at these particular inpatient units provided patients with the knowledge to self-manage their fall risk from day one of rehabilitation.

Community passes were recognized as a way to prepare patients for the risk of falls in the community. Administrators explained that home visits were conducted with most inpatients to provide patients with additional information on the fall hazards in their post-discharge environment. According to administrators, it was difficult to prepare patients for fall risk in the home environment because the recommended level of assistance and funding for appropriate equipment may not be widely available outside a hospital setting.

#### 3c) Non-preventable falls

Administrators discussed many scenarios where they felt they were not able to prevent falls. For instance, some falls that were believed to be a result of the “patient’s own actions” were considered non-preventable. Impulsive behavior and non-adherence with safety recommendations were examples of patient-related challenges to fall prevention. One proposed solution for these challenges was to tailor fall prevention education to match the personality and behaviour of the patient.

Another challenging situation where administrators felt they could not prevent falls was when a patient leaves the facility. A3 explained, “it’s out of our control once they leave the building in terms of if a fall happens, but you know we still feel accountable because they are our patients” (A3). Finally, some administrators believed that non-preventable falls were seemingly inevitable due to the nature of SCI. As A9 stated, “I have an incomplete spinal cord injury patient, where a patient could be walking with two staff and we have everything in place but a leg could buckle”. As such, their efforts were shifted to minimizing injuries from the fall.

## Discussion

**Fig. 1:**
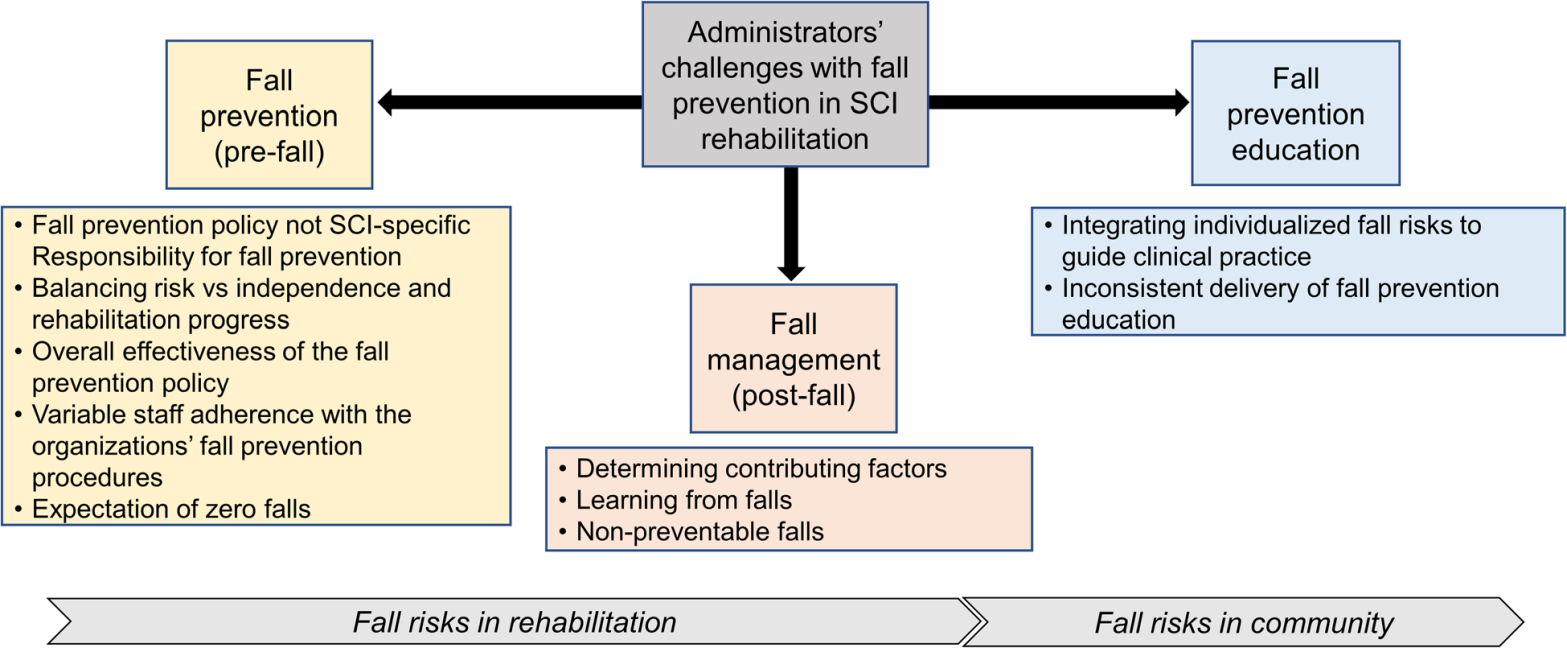
Overview of components of fall prevention in Canadian tertiary rehabilitation hospitals

This is the first study to examine the perspectives of hospital administrators regarding implementation challenges with fall prevention in Canadian SCI rehabilitation. It is important to consider that SCI rehabilitation in Canada operates within a publicly funded healthcare system. Fall prevention was considered a high priority for administrators of all hospitals. Findings from this study uncovered three categories of challenges that administrators faced while they implemented fall prevention in SCI rehabilitation: policy and procedural, clinician-related, and patient-related. These challenges impact fall prevention and management across rehabilitation and community environments (see Fig. 1). In the current study, similar fall prevention challenges were reported by administrators from both SCI-specific units as well as general rehabilitation units. This may be because the fall prevention policies implemented at all sites were generic and not population specific to individuals with SCI.

Presented in Table 3 are a list of recommendations for SCI rehabilitation within tertiary rehabilitation hospitals that may address challenges raised by administrators. These recommendations are informed by findings from this study, as well as preceding studies [12, 14, 29–32].

**Table 3:** Clinical practice recommendations

Fall Risk Assessments in SCI rehabilitation	
■ Re-format fall risk assessment tools to encourage critical thinking and individualized plans	
■ Consider a patient’s behavioral risk factors to predict fall risk	
Fall Prevention Procedures in SCI rehabilitation	
■ No blame reporting of fall incidents	
■ Consider strategies to minimize injuries from falls	
■ Establish consensus on documenting therapy falls that do not result in an injury and streamline reporting process	
■ Adapt existing fall prevention strategies to increase applicability in SCI rehabilitation	
■ Track falls during community passes to identify areas for additional fall prevention education	
■ Re-design processes that have poor compliance	
Fall Prevention Training/Education in SCI rehabilitation	
■ Include SCI-specific fall risk factors when delivering fall prevention education	
■ Prioritize fall prevention education in inpatient and outpatient rehabilitation	
■ Educate patients with SCI on hospital and community fall risk factors	
■ Include an informed risk taking and/or self-management approach to fall prevention training	
■ Create a formal dialogue for fall prevention education	
■ Ensure clinicians have the appropriate skills to deliver fall prevention education	
Post-fall Procedure in SCI rehabilitation	
■ Conduct formal reviews of fall trends and integrate findings into fall prevention education	
■ Share fall trends with staff and encourage discussions on how to prevent similar occurrences	
■ Identify the patients’ perspectives on contributing factors to falls	
■ Leadership to continue encouraging a culture of learning from falls	
■ Outline a clear process for clinicians to follow if a patient refuses the post-fall assessment	

One challenge identified by the administrators was accurately predicting fall risk in patients with SCI. Consistent with prior research [14, 29], administrators acknowledged that there was a lack of screening tools to accurately predict falls in rehabilitation. Despite fall prevention best practices and Accreditation Canada requiring the use of a risk assessment tool to predict a patient’s risk of falling [1, 3], administrators believed most tools currently being used for patients with SCI in tertiary rehabilitation hospitals lacked the ability to discriminate between those at low and high risk of falling. This may be because most fall risk assessment tools have not been developed or validated in a rehabilitation setting [33]. Administrators found some rehabilitation units had low staff adherence with completing the risk assessment tool. We found some of the administrators themselves did not buy into all tools mandated by the organization for their units. As a result, these administrators struggle with enforcing a process which they themselves feel has little utility in this specific setting. This finding signifies a disconnect between the organization’s fall prevention requirements and the clinical realities of working with patients with SCI in tertiary rehabilitation hospitals that should be addressed.

Given the limited options of risk assessment tools appropriate for patients with SCI in rehabilitation, it would be worthwhile to develop sensitive methods of evaluating fall risk in this population. For example, a study from a geriatric rehabilitation unit found clinical judgment to have higher accuracy in predicting falls than risk assessment tools [29]. Aizen & Zlotver, (2013) found developing a single fall risk prediction tool to use with multiple populations in rehabilitation settings was challenging due to the multifactorial causes of falls. Instead, checklists that prompted clinicians to consider common population-specific fall risk factors may have greater clinical utility than a fall risk assessment in a rehabilitation setting [17]. Rather than adopting the same assessment tools used in acute care, rehabilitation hospitals should determine which fall risk assessment tool is most appropriate for assessing patients’ risks of falling within their setting [30, 34].

Post-fall debriefs offer a chance to learn from a fall event, identify risk factors that cause falls, and can inform specific interventions for a patient [31]. All administrators in this study described the occurrence of a post-fall follow-up, but a larger review of the ongoing trends was lacking. Since a regular evaluation of the effectiveness of a healthcare organization’s fall prevention program is a required organizational practice [1], this finding highlights a practice gap. A more formalized review of the effectiveness of the fall prevention policy and procedures, fall trends and subsequent practice recommendations at each participating unit would be beneficial.

It was the collective standpoint of administrators that striving for zero falls when working with patients with SCI in rehabilitation was not a realistic goal. Cochrane & colleagues (2017) suggest that zero patient harm may be a more appropriate goal in rehabilitation [35]. Administrators discussed rehabilitation as a place where patients were going through the journey of relearning their physical abilities, and regaining their mobility and autonomy. Consistent with previous study, the challenge of balancing the autonomy and the safety of patients with SCI during their rehabilitation remains a major challenge in fall prevention [30]. In cases where no harm occurred, administrators viewed these falls as learning opportunities for both the patient and clinicians. In SCI rehabilitation, the aims are to achieve goals related to motor tasks including ambulation, wheelchair propulsion, and transfers [36]. These motor tasks are less likely to result in injury because they are practiced under the supervision of a trained therapist [37]. As such, in SCI rehabilitation, injurious falls may be a more appropriate quality measure, while no-harm falls and near falls can be approached as learning opportunities [38].

Hospital falls are classified as adverse events, defined as “an unintended injury or complication resulting in prolonged hospital stay, disability at the time of discharge or death and caused by healthcare management rather than by the patient’s underlying disease process” [39]. This suggests a notion that all hospital falls are preventable [38]. In contrast to this view, we found administrators believed that many falls were a result of intrinsic SCI complications and consequently not preventable. Administrators also faced challenges with preventing falls in patients who demonstrated risk taking or impulsive behaviour. Fall prevention studies have recognized the role of behavioural factors such as a person’s decision making and a person’s risky behaviour as contributors to falls [33, 40]. Informed risk taking is one approach that clinicians can use to educate patients who exhibit impulsive or risk taking behaviour. This approach involves informing and educating all patients on their fall risk factors and encouraging patients to consider their risk of falling with all activities that they will engage in. As patients participate in rehabilitation and learn their new abilities, their “risk taking” will be informed, voluntary, and practiced in a supportive environment [41].

We found some administrators lacked awareness of the high rate of falls faced by people with SCI after discharge from rehabilitation [42]. Knowledge gaps regarding the prevalence of falls after SCI may lead to a lower prioritization of fall prevention education/training in SCI rehabilitation. Moreover, some administrators had not considered the need to transfer the responsibility of fall prevention from hospital staff to the patient. Only two administrators viewed fall prevention as a shared responsibility between the patient and clinicians and encouraged a self-management approach to fall prevention from day one following admission to rehabilitation. Using this approach, patients were educated on their fall risks and then encouraged to identify what strategies to use to prevent falls. Even though this approach was not implemented on all units, teaching self-management skills for fall prevention was generally positively perceived by administrators and thought to make patients more prepared for community fall risk factors. A self-management fall risk program in multiple sclerosis has been found to improve skills and behaviour that can reduce the risk of falls [43].

The strengths of this study are the representation of interdisciplinary perspectives of administrators from six tertiary hospitals, who are often difficult to reach. Telephone interviews allowed us to include administrators who provide services to patients with SCI from tertiary rehabilitation hospitals across Canada; however, the absence of visual cues in their telephone interviews may have led to misunderstandings [44] and a loss of engagement between the interviewee during the conversation is a limitation of this study [45].

## Conclusion

This study revealed implementation challenges related to fall prevention from the perspectives of hospital administrators who provide services to patients with SCI in a tertiary rehabilitation hospital. Challenges related to three categories were identified including policy and procedural, clinician-related and patient-related. The generic organizational fall prevention policies and procedures were found to be insufficient for addressing the clinical realities of fall prevention that takes place within SCI rehabilitation. Findings highlight ways that comprehensive fall prevention education can facilitate the transfer of fall prevention responsibility from hospital staff to administrators during the discharge transitions from SCI rehabilitation.

## Additional file

Additional file 1: Interview guide. (DOCX 25 kb)

## Data Availability

The datasets generated and/or analysed during the current study are not publicly available due confidentiality of the transcripts but are available from the corresponding author on reasonable request.

## Abbreviations

SCI: Spinal cord injury
UHN: University Health Network
