# Supplementary material for: Factors influencing fall prevention for patients with spinal cord injury from the perspectives of administrators in Canadian rehabilitation hospitals
Source: BMC Health Serv Res. 2019 Jun 17;19:391. doi: 10.1186/s12913-019-4233-8 (PMC6580568; doi:10.1186/s12913-019-4233-8)
Supplement: Supplementary file 1 — Interview guide. (DOCX 25 kb) [file 12913_2019_4233_MOESM1_ESM.docx]

**Interview guide**

This study is being conducted to explore how administrators understand systems level challenges and opportunities to prevent and manage falls in spinal cord rehabilitation units. There are no right or wrong answers, so please feel free to share anything that comes to mind on this topic.

**Hospital Falls**

1. Tell me about your experiences with Fall Prevention and Management Strategies/ Programs on the SCI unit.
2. How does the SCI unit define a fall?
   1. Is the definition of a fall different in the outpatient vs inpatient units?
3. What does a good SCI unit’s Fall Prevention and Management Strategy or Program look like?
4. How would you measure success of the fall prevention/management strategy on a SCI unit?
5. Could you describe the process of how the Fall Prevention and Management Strategy or Program are created for the SCI unit?
   1. Which guidelines/protocols are followed?
6. Could you tell me about your SCI unit’s Fall Prevention and Management Policy, Strategies or Programs on the SCI unit?
   1. Is it effectiveness?
   2. What concerns do you have about the policy? The strategy or programs?
   3. What is working well?
   4. What is the best thing that has happened over the last few years to the SCI unit’s fall prevention policies or strategy/programs?
   5. What could be improved?
7. Tell me about the staff/patient culture on the SCI unit with respect to falls.
   1. How could it be improved?
   2. How compliant are staff with the fall prevention/tracking?
   3. How does your SCI unit’s Fall Prevention and Management Strategies or Programs impact clinical practice?
8. How are falls classified on the SCI unit?
   1. What are the different classifications for falls? (i.e. preventable vs not preventable; major vs minor injury
   2. Is this classification of falls effective?
   3. What are some of your concerns about the classification system for falls?
9. What challenges do you face when tracking falls?
   1. How could falls tracking be improved?
10. Could you describe what steps happen after a patient has a fall on the SCI unit?
    1. Who is consulted?
    2. What management steps are taken to prevent future falls?
11. To what extent do you think falls are an issue with individuals with SCI while in inpatient and outpatient rehabilitation?
    1. What causes falls in hospitals?
12. Could you identify three most significant challenging facing Fall Prevention and Management Strategies or Programs on the SCI Unit?
    1. How could these challenges be improved?
13. What is a reasonable target for fall rates?
    1. What is your opinion on "Zero Falls" aims?
14. What resources are available for the clinical staff (i.e. nurses, physicians, therapists) to prevent and reduce patient falls on the SCI unit?

**Community Falls**

1. What is your understanding of falls in the community for people with SCI?
   1. What percentage of individuals with SCI have a fall after discharge?
2. Why do some fall when they are back in the community?
   1. What is different about those who do not fall when they’re back in the community?

*Facilitator: 75% of ambulatory individuals with SCI experience at least one fall (Brotherton, Krause, & Nietert, 2007), while 64% of non-ambulatory wheelchair users with SCI experience a fall in the community (Butler Forslund et al., 2017).*

1. Given this statistic, what role, if any, does the SCI Unit play in minimizing falls both in the hospital and after individuals with SCI return home from the hospital?
   1. In what ways do hospitals play a role to minimize falls after individuals with SCI return home from the hospital?
   2. What are challenges for hospitals around falls when someone leaves the hospital?

Literature shows the transitions from hospital to home involve a “transfer of responsibility” during which the responsibility for controlling the health and well-being of the patient moves from the hospital to the caregivers/patients which take on primary responsibility for participation in fall prevention activities (Hill et al., 2017).

1. What are your thoughts on transfer of fall management responsibility? What are your thoughts on how well the SCI unit here facilitates care transitions?
2. To what extent is it a priority to reduce falls in the community on the SCI Unit?
   1. How best could this occur?
3. To what extent do the inpatient and outpatient Fall Prevention and Management Strategy, Program or Approaches in the SCI Unit focus on prevention of falls after discharge from hospital for people with SCI? Please describe.
4. How can the outpatient SCI unit Fall Prevention and Management Strategy, Program or Approaches include fall prevention after individuals leave the hospital?
   1. What concerns do you have about this?
   2. What are some positives about this?
5. In your opinion, are there any opportunities in the hospital to prepare patients with SCI to return to living in the community?
   1. What would this look like? Where is the best place to meet this goal?
   2. What other important issues should you consider when creating or modifying current fall policies/strategies/approaches?

**Closing**

1. Is there any additional information or final thoughts on this topic that you would like to add?
